# Beyond calories: the effect of meal patterns on obesity risk in the IRanian National Obesity Registry

**DOI:** 10.34172/jcvtr.026.33600

**Published:** 2025-12-17

**Authors:** Mina Nosrati, Niosha Samadi, Ali Mottaghi Moghadam Shahri, Mahsa Tousi, Farnaz Farrokhzadeh, Fateme Kourepaz, Najmeh Seifi, Khalil Kimiafar, Majid Ghayour Mobarhan

**Affiliations:** ^1^International UNESCO Center for Health-Related Basic Sciences and Human Nutrition, Mashhad University of Medical Sciences, Mashhad, Iran; ^2^Department of Nutrition, Faculty of Medicine, Mashhad University of Medical Sciences, Mashhad, Iran; ^3^Department of Nutrition Sciences, Varastegan Institute for Medical Sciences, Mashhad, Iran; ^4^Department of Medical Records and Health Information Technology, School of Paramedical Sciences, Mashhad University of Medical Sciences, Mashhad, Iran

**Keywords:** Obesity, BMI, Waist circumference, Breakfast, Meal skipping

## Abstract

**Introduction:**

The prevalence of obesity is increasing. Eating habits are modifiable factors that may be effective in reducing obesity. We aimed to investigate the relationship between meal frequency and daily breakfast and the presence of obesity.

**Methods:**

A total of 4137 overweight and obese individuals registered on IRanian National Obesity Registry (IRNOR) in 2022 were recruited into this cross-sectional study. Individual data was recorded using a self-reported questionnaire. Multivariate logistic and linear regressions were used to evaluate the association between meal frequency, daily breakfast and obesity.

**Results:**

There was a lower significant odd of obesity (odd ratio (OR)=0.80, 95% confidence interval (CI): 0.69-0.93; *P*=0.004) in people who reported having more than three meals a day. There was a lower odd of obesity (OR=0.79, 95%CI: 0.69-0.91; *P*=0.001) in individuals who had daily breakfast and higher odds of obesity (OR=1.21, 95%CI: 1.03-1.42; *P*<0.01) for individuals who reported dinner as their main meal of the day. There was a significant association between eating frequency and daily breakfast with central obesity in men and in women; there was a significant inverse relationship between eating breakfast and central obesity.

**Conclusion:**

Meal frequency and eating breakfast daily as a modifiable eating habits can lead to improvement obesity.

## Introduction

 The prevalence of overweight and obesity has been increasing globally, and it is reported to be a pandemic.^1^ The global prevalence of overweight and obesity has risen sharply, reaching over 2.1 billion adults in 2021. The most rapid increases occurred in the Middle East and north Africa, where prevalence in men more than tripled and in women more than doubled since 1990. Projections estimate that, by 2050, over half of the world’s adult population(~3.8 billion people) will be overweight or obese.^2^ In Iran, recent meta-analyses report that the overall prevalence of obesity is approximately 10.9%, and among adults, it reaches around 17.2%.^3^ Moreover, a 2025 population-based study in Fars province found that overweight and obesity prevalence among primary school children was 26.5%, including 9.4% classified as obese. ^4^ An estimated 2.8 million people die each year from obesity-related complications, including cardiovascular disease, metabolic syndrome and type 2 diabetes.^5^

 Obesity is a multifactorial disease influenced by sedentary lifestyle and dietary habits, genetic susceptibility, social and psychological factors.^1^ The first-line therapy of obesity is lifestyle and the modification of eating habits. Eating habits including meal frequency, speed of eating and skipping breakfast have potential effects on human health.^6^ A higher meal frequency has previously been related to an improvement in lipid profile, reduction in fat mass and lower risk of obesity and cardiovascular disease and type 2 diabetes.^6-10^ Regular breakfast consumption is inversely associated with central and general obesity, potentially through mechanisms involving appetite control, improved satiety, and metabolic regulation.^11^ Evidence also suggested that late eating dinner is associated with higher body mass index (BMI), unfavorable metabolic profiles, and reduced weight-loss success, while earlier meal timing-independent of total energy intake and physical activity-leads to greater improvements in weight, adiposity, and metabolic risk markers.^12,13^ However, some other studies have not found the same association.^14,15^

 Similarly, epidemiological studies have also reported skipping breakfast is associated with higher risk of obesity.^10,16^ These findings are in contrast with other studies that have reported no association between meal frequency and eating breakfast with obesity, fat mass, diabetes and coronary heart disease and despite the numerous studies in this regard the results are controversial and raise questions about diversity of population, methodological approaches and meal content.^10,14,15,17,18^

 Given these alarming trends in obesity-particularly among children and adolescents- and the limited number of investigations into modifiable lifestyle factors such as meal frequency and breakfast consumption,^3,16^ along with cultural eating habits in Iran that are likely to differ from Western populations, we aimed to examine the relationship between meal frequency and breakfast consumption in the IRanian National Obesity Registry (IRNOR) population.

## Materials and Methods

 This was a cross-sectional, multi-center and population-based study derived from IRNOR, which was started in 2022 in Mashhad, Iran. The registry questionnaire was self-reported and validated using the Delphi technique^19^ in two rounds with the participation of experts in nutrition and health information technology. Subsequently, a web-based registry platform was developed (www.angabinteb.com). Afterwards, a call was announced for centers interested in participating in the obesity registry. Data were collected from several cities including, Mashhad and Ghaemshahr cities, two private clinics in Hamedan and Mashhad and public healthcare center in Mashhad. Data validation was performed using the data dictionary, and the data were checked periodically and will be rechecked before any analysis. The data of 4,173 overweight and obese individuals who aged over 18 years old were recorded. Demographic, anthropometric, medical history, physical activity, eating habits, and smoking status were collected on registry website through a face-to-face interview by trained professionals. The inclusion criteria were age ≥ 18 years and BMI ≥ 25 kg/m^2^, and those unwilling to participate were excluded.An electronic informed consent form was placed at the beginning of the registry questionnaire, and participants were enrolled in the registry only after reading and confirming their consent.

 This study was approved by the ethics committee of Mashhad University of Medical Sciences (IR.MUMS.MEDICAL.REC.1401.038).

###  Demographic and lifestyle data

 Age, sex, marital status, employment, education, and smoking status were recorded using a self-reported questionnaire.

###  Measurements

 Anthropometric measurements, including weight (kg), height (cm), waist circumference (WC) (cm), fat mass (kg), BMI (kg/m^2^), systolic (mmHg) and diastolic (mmHg) blood pressure, were measured by a trained professional with standard protocol.^20,21^ According to World Health Organization (WHO) criteria, 18.5 ≤ BMI < 24.9 was defined as normal, 25 ≤ BMI < 29.9 as overweight and BMI ≥ 30 as obesity in adult. ^22^ Central obesity was defined as WC ≥ 90 in men and WC ≥ 80 in women, according to International Obesity Task Force central obesity criteria for Asia.^23^

###  Physical activity level (PAL)

 We used the short form of self-administered International Physical Activity Level Questionnaire (IPAQ) to assess PAL.^24^ According to the IPAQ, physical activity, including sedentary behavior, walking, moderate and vigorous activity, was evaluated over the last seven days or based on usual activity.^24^

###  Meal frequency Assessment

 Meal frequency and main meal were evaluated using structured questions developed and validated during the IRNOR project through a Delphi process involving 21 nutrition experts:

 How many meals and snacks do you eat daily? (“1”,”2”, “3”, “4”, “5”, “more than 5”)

 Your main meal is: (“Breakfast”, “Lunch”, “Dinner”)

###  Statistical Analysis

 We usedthe Statistical Package for the Social Science version 26.0 (SPSS, Chicago, IL) to perform statistical analysis. Categorical and continuous variables were described by percent and mean ± standard deviation respectively. To evaluate the difference between groups by meal frequency we used one-way ANOVA test for continuous variables and Chi-Square test for categorial variables. Multivariate logistic regression was used to evaluate the association of meal frequency and eating breakfast with obesity (BMI ≥ 30). We used multivariate linear regression used to assess the association of meal frequency and eating breakfast with central obesity by gender. The adjustment was done for age, gender, job, PAL, marital and smoking according to previous studies.^16,25^ A *p*-value < 0.05 was considered significant.

## Results

Table 1 shows the characteristics of 4173 Iranian adults categorized by 3 meal frequency into three groups: < 3 meals, = 3 meals and ≥ 3 meals daily.

**Table 1:** Baseline characteristics of participants stratified by meal frequency categories

**variables**		**Meal frequency**									***P*** **-value**
		**<3** **N=656**			**=3** **N=2244**			**>3** **N=1273**			
		**%**	**Mean **	**SD**	**%**	**Mean **	**SD**	**%**	**Mean **	**SD**	
Gender	Male Female	29.7 70.3			32.8 67.2			40.3 59.7			< 0.0001
Age (year)	19-29 30-49 ≥ 50	23.1 14.4 10.5			48.6 54.5 58.1			28.3 31.2 31.4			< 0.0001
BMI (kg/m^2^)	< 30 ≥ 30	47.9 52.1			48.4 51.6			56.6 43.4			< 0.0001
Married Single & divorce		72.7 27.3			82.0 18.0			80.1 19.9			< 0.0001
Smoking	Yes No	11.0 89.0			5.6 94.4			7.2 92.8			< 0.0001
Employment Student & unemployed Housewife Retired		50.2 14.4 33.2 2.2			52.0 8.5 33.9 5.6			61.2 8.1 25.8 4.9			< 0.0001
Illiterate up to secondary school Under graduated Post graduated		14.5 34.8 50.7			15.0 34.1 51.0			17.1 31.7 51.2			< 0.35
PAL	No activity Walking Moderate Severe	63.5 16.7 17.0 2.8			56.9 19.3 18.4 5.4			44.6 24.2 22.6 8.6			< 0.0001
Main meal	Breakfast Lunch Dinner	6.0 13.9 39.2			52.3 54.5 40.5			41.7 31.6 20.3			< 0.0001
Diabetes mellitus (yes) Hypertension (yes) Dyslipidemia (yes)		2.7 5.3 4.0			6.7 7.8 5.1			8.5 10.4 6.8			< 0.0001 < 0.0001 0.017
WC (cm)			101.04	15.39		100.78	13.88		99.58	12.91	< 0.0001
SBP (mmHg)			124.07	15.78		124.62	16.46		123.43	16.79	< 0.0001
DBP (mmHg)			81.69	12.89		80.77	11.85		80.61	11.39	< 0.0001

BMI: body mass index; PAL: physical activity level; WC: waist circumference; SBP: systolic blood pressure; DBP: diastolic blood pressure

 The mean ages between groups ( < 3 meals, = 3 meals and ≥ 3 meals) were 35.52, 40.26 and 40.21 years respectively and there was a significant difference between groups (*P* < 0.0001). The percent of female was higher than males in all groups (70.3% in < 3 meals group, 67.2% in = 3 meals and 59.7% in ≥ 3 meals) (*P* < 0.0001).

 The percentage of participants who had < 3 meals was higher in BMI ≥ 30 (52.1%) compared to other groups (3 meals: 51.6%) ( > 3 meals: 43.4%) (*P* < 0.0001).

 Lifestyle factors such as physical activity, smoking, job and marital status also differed significantly between groups.

Table 2 represents the relationship between meal frequency and BMI ≥ 30. The group with three meals was considered as a reference group. In model 1, individuals with more than three meals had 27% lower odds of BMI ≥ 30 (odd ratio (OR) = 0.73, 95% confidence interval (CI): 0.63 - 0.84; *P* < 0.0001) when adjusted for age and gender. In model 2, this association was remained after adjustment for job, PAL, marital status and smoking (OR = 0.80, 95%CI: 0.69-0.93; *P* = 0.004).

**Table 2:** Association between meal frequency and BMI ≥ 30 by multivariate logistic regression

	**Meal frequency**					
	**<3** **N=656**		**=3** **N=2244**	**≥3** **N=1273**		
	**OR**	**95%CI**		**OR**	**95%CI**	***P*** **-trend**
Crude	1.02	0.85- 1.21	1	0.71	0.62 -0.82	< 0.0001
Model	1.04	0.88-1.25	1	0.73	0.63-0.84	
Model2	1.00	0.82-1.21	1	0.80	0.69-0.93	

Model1: Adjusted for age & gender Model 2: Adjusted for model1 + job, PAL, marital, smoking.

Table 3 demonstrates the association between the consumption of breakfast and dinner as main meals and BMI ≥ 30. In model 1 participants with breakfast as a main meal had lower odds of BMI ≥ 30 when adjusted for age and gender (OR = 0.66, 95%CI: 0.58-0.74; *P* < 0.0001). This association was remained in Model 2 when adjusted with job, PAL, marital and smoking (OR = 0.79, 95%CI: 0.69-0.91; *P* = 0.001). According to this table having dinner as a main meal had 22% higher odds of BMI ≥ 30 (OR = 1.22, 95%CI: 1.05-1.43; *P* < 0.0001) in model 1 and (OR = 1.21, 95%CI: 1.03-1.42; *P* < 0.01) in model 2 after adjustment for confounder factors.

**Table 3:** Association between main meal and BMI ≥ 30 by multivariate logistic regression

**Main meal**	**BMI≥30**		
**Breakfast ** **N=1857**	**OR**	**95%CI**	***P*** **-value**
Crude	0.66	0.58- 0.74	< 0.0001
Model 1	0.66	0.58-0.74	< 0.0001
Model 2	0.79	0.69-0.91	0.001
Dinner N = 837	OR	95%CI	*P*-value
Crude	1.18	1.01- 1.37	0.03
Model 1	1.22	1.05-1.43	0.009
Model 2	1.21	1.03 – 1.42	0.01

BMI: body mass index Model 1 adjusted for age & gender Model 2: Adjusted for model 1 + job, PAL, marital, smoking.

Table 4 indicates the relationship of meal frequency and eating breakfast with central obesity by gender. Among men, higher meal frequency and breakfast consumption were significantly and inversely associated with central obesity. Increased meal frequency decreased waist circumference (B = -2.35, *P* < 0.0001) and eating breakfast regularly had a similar effect (B = -3.45, *P* < 0.0001) after adjustment for confounder factors. Among women, the associations between eating breakfast and central obesity were significant (B = -1.27, *P* = 0.01), but this association in meal frequency was not significant (*P* = 0.93) after adjustment.

**Table 4:** Multivariate linear regression analysis of meal frequency and breakfast consumption with central obesity, stratified by gender

	**Men**			
	**B** **(unstandardized)**	**SE**	**β (standardized)**	***P*** **-value**
Meal frequency	-2.35	0.54	-0.13	< 0.0001
Eating breakfast	-3.45	0.75	-0.14	< 0.0001
Eating dinner	2.48	0.87	0.08	< 0.0001
	**Women**			
	B	SE	β (standardized)	*p*-value
Meal frequency	0.02	0.36	0.002	0.93
Eating breakfast	-1.27	0.49	-0.05	0.01
Eating dinner	0.61	0.61	0.02	0.32

ES: standard Error Adjusted for age, job, PAL, marital, smoking.

## Discussion

 Our findings indicated that there is a negative association between higher meals frequency and eating breakfast regularly with BMI and central obesity, while eating dinner as a main meal was correlated to higher BMI and central obesity just in men after further adjustment. These results are consistent with previous epidemiological studies that demonstrated protective effects of increased meal frequency and breakfast intake against obesity.^11,26^

 Similar to our findings, several previous studies have reported this negative association of more than three meals frequency and eating breakfast regularly and positive association of eating dinner as a main meal with obesity.^11,16,27-29^ Recent evidence in chrono-nutrition emphasizes that not only “what we eat,” but also “when we eat” plays a crucial role in metabolic health. Misalignment between meal timing and circadian rhythms may impair glucose regulation, insulin sensitivity, and energy expenditure, thereby promoting fat accumulation. Individuals who consume their meals later in the day have poorer glucose tolerance and reduced weight loss, whereas earlier meal timing is associated with more favorable outcomes.^30,31^ However, some studies have reported no significant associations,^32,33^ possibility because of their small sample size, diversity in population, different in baseline BMI, demographics and quality of dietary intakes and different assessment methods. In addition, previous studies have shown that dietary patterns vary between breakfast, lunch, and dinner among the Iranian population. It was reported that breakfast meals were higher in cheese, bread, and vegetables, similar to the Mediterranean diet, while characteristics of the Western dietary pattern were observed in lunch and dinner meals in the Iranian population.^34^ Dietary patterns may differ based on sex, ethnicity, socioeconomic factors, food security, employment status, educational level, and marital status across different populations. Furthermore, variations in religion, culture, and beliefs can influence the diversity of dietary patterns.^34-37^

 One of the possible underlying mechanisms for this association is variations in glucose concentrations. More frequent meals during the day may decrease the postprandial spikes of insulin and improve stability of blood glucose that reduce fat accumulation.^38^ In contrast, fewer meal frequency consequence of prolonged fasting periods may promote overeating at meal times. Another potential mechanism is hunger sensation. Eating more frequently leads to reduced overall energy intake through decreasing hunger sensation.^39^ However, more frequent meals with high caloric meals and snacks can lead to weight gain. In this study we have no dietary intake of participants and should be considered in further studies.

 Skipping breakfast may promote weight gain, insulin resistance, metabolic risk, atherosclerosis and cardiovascular disease independent of amount of calorie intake through effects on the circadian rhythm. Skipping breakfast changes appetite and reduces satiety which can cause overeating and insulin sensitivity impairment. While, eating breakfast can breakdown prolonged overnight fasting, decrease ghrelin, regulate appetite.^40,41^ Moreover, daily breakfast might increase 24-hour energy expenditure, which can lead to prevent weight gain.^42^ People with irregular or skipping breakfast usually have unhealthy behaviors including, smoking, sedentary lifestyle and unhealthy food choices.^28^ Our findings are in line with this evidence, as breakfast skippers in our study were more likely to be smokers and physically inactive.

 This study has several strengths, including the use of a large multicenter sample from different regions of Iran, application of standardized and validated tools for data collection, and statistical adjustment for multiple potential confounders. These strengths enhance the generalizability and robustness of our findings. This study has several limitations. First, its cross-sectional design precludes establishing causal relationships between meal frequency, breakfast consumption, and obesity. Second, reliance on self-reported eating habits may have introduced recall or reporting bias, thereby affecting the accuracy of the data. Third, the study population may not be fully representative of other cultural or ethnic groups, which may limit the generalizability of the findings. Future research employing longitudinal designs and detailed dietary intake assessments is recommended to validate and extend these results.

## Conclusion

 In conclusion, we have reported the strong positive significant association of eating meal frequent and breakfast as a main meal with obesity and central obesity in Iranian obese adults. These findings help clinicians consider eating behaviors in obesity treatment and design personalized interventions.

## Competing Interests

 The authors declare no conflict of interests.

## Ethical Approval

 This study was approved by the ethics committee of Mashhad University of Medical Sciences (IR.MUMS.MEDICAL.REC.1401.038). All participants gave informed consent.
